# Rapid identification and management of stress-induced cardiomyopathy using POCUS after strangulation: A case report

**DOI:** 10.1097/MD.0000000000039532

**Published:** 2024-09-13

**Authors:** Juho An, Sung-Eun Lee

**Affiliations:** a Department of Emergency Medicine, Ajou University School of Medicine, Suwon, Republic of Korea.

**Keywords:** cost effectiveness, hanging, POCUS, SCMP, Takotsubo syndrome

## Abstract

**Rationale:**

Stress-induced cardiomyopathy (SCMP), also known as Takotsubo syndrome, is a transient cardiac condition often precipitated by severe emotional or physical stress. It is commonly mistaken for acute coronary syndrome due to similar clinical presentations. The use of point-of-care ultrasound (POCUS) provides a noninvasive, rapid diagnostic alternative that can potentially reduce the need for invasive coronary angiography, especially in emergency settings.

**Patient concerns:**

A 26-year-old woman with type 1 diabetes presented to the emergency department following a suicidal hanging attempt. Upon arrival, she was conscious but confused, with stable vital signs. There were visible signs of strangulation, but no other immediate physical abnormalities. Laboratory tests revealed elevated cardiac enzymes and hyperglycemia.

**Diagnoses:**

Initial bedside POCUS revealed a reduced ejection fraction and regional wall motion abnormalities in the midportion of the left ventricle, suggesting SCMP. These findings, combined with the patient’s history and absence of other contributory factors, led to a provisional diagnosis of SCMP.

**Interventions:**

The patient was admitted to the intensive care unit for close monitoring. Serial POCUS examinations were performed to track cardiac function. Due to the rapid improvement in regional wall motion abnormalities observed through POCUS, the planned coronary angiography was deferred.

**Outcomes:**

The patient exhibited significant clinical improvement within 24 hours, with normalization of cardiac function as demonstrated by follow-up POCUS. Cardiac enzyme levels also returned to normal. The patient was discharged directly from the intensive care unit without the need for further invasive procedures.

**Lessons:**

This case underscores the diagnostic value of POCUS in rapidly identifying SCMP in emergency settings, which can guide timely and appropriate management. The noninvasive nature of POCUS may reduce the need for invasive diagnostics, minimize hospital stay duration, and enhance cost-effectiveness in managing SCMP.

## 1. Introduction

Stress-induced cardiomyopathy (SCMP), also referred to as Takotsubo syndrome, is a condition in which severe stress leads to temporary heart dysfunction. It is often mistaken for myocardial infarction because of similar symptoms, but SCMP does not involve coronary blockage.^[1–3]^ Diagnosis traditionally requires invasive methods such as coronary angiography to differentiate it from other coronary diseases.^[1,3]^ This report focuses on a case of SCMP after strangulation that demonstrates the role of point-of-care ultrasound (POCUS) in emergency and critical care settings. Owing to its rapid and noninvasive diagnostic capability,^[4,5]^ POCUS can be used to differentiate SCMP from more severe cardiac diseases and to guide immediate management decisions. POCUS reduces the need for invasive procedures, thereby minimizing patient discomfort and the risk of complications. The case described herein highlights the essential role of this procedure in modern emergency medicine, as well as its efficiency in managing conditions such as SCMP.

## 2. Case report

A 26-year-old woman with a history of type 1 diabetes was found unconscious in a motel room with a rope around her neck and transported to the emergency department (ED) via ambulance. Upon arrival, the patient showed symptoms of confusion. At that time, her vital signs were stable: oxygen saturation, 100%; blood pressure, 131/83 mm Hg; pulse rate, 100 beats/min; and respiratory rate, 26 breaths/min. Physical examination revealed traces of rope around the patient’s neck, and there were no unusual findings pertaining to the lungs, heart, or abdomen.

**Figure 1.**
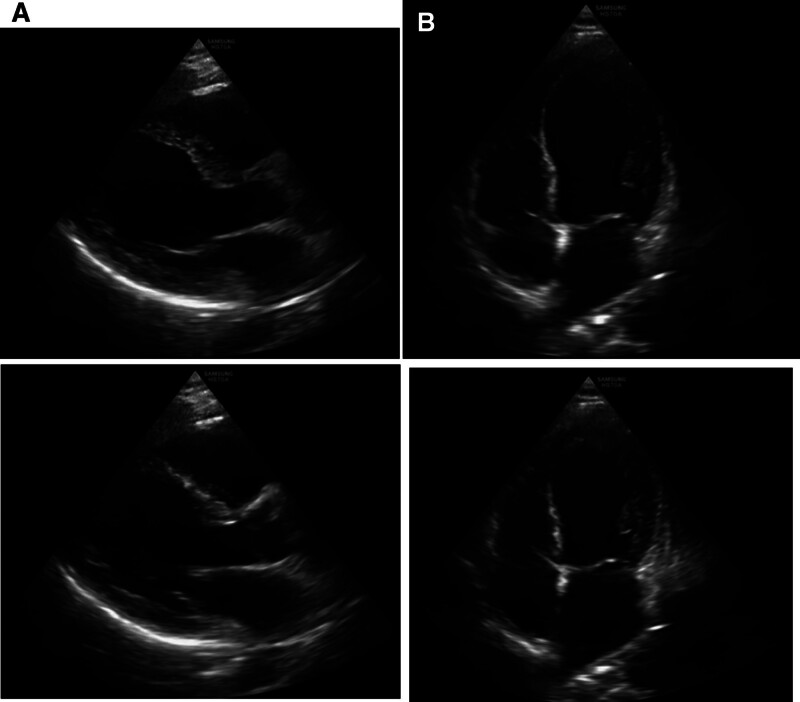
Initial cardiac POCUS of the end-diastolic and end-systolic phases shows regional wall motion abnormality at midportion of left ventricle. (A) Parasternal long-axis view of the end-diastolic (upper) and end-systolic (lower) phases. (B) Apical four-chamber view of the end-diastolic (upper) and end-systolic (lower) phases.

Laboratory tests revealed an elevated white blood cell count (WBC) of 20,900, a hemoglobin (Hb) level of 15.5 g/dL, a hematocrit (Hct) test result of 46.9%, and a platelet count (PLT) of 484,000. Biochemical analysis revealed no abnormalities except for hyperglycemia (glucose, 594 mg/dL; HbA1c, 13.4%). Cardiac markers showed creatine kinase (CK) levels within the normal range at 129 U/L (reference range: 26–192 U/L), but CK-myocardial band (CK-MB) and high-sensitive Troponin T (hs-TnT) levels were elevated at 6.22 μg/L (reference range: 0.0–5.0 μg/L) and 0.060 ng/mL (reference range: 0.000–0.014 ng/mL), respectively. Lactic acid was also elevated at 4.62 mmol/L (reference range: 0.7–2.0 mmol/L). The initial arterial blood gas analysis indicated the following: pH: 7.510, pCO_2_: 20.6 mm Hg, pO_2_: 101.8 mm Hg, and HCO3: 16.5 mmol/L. An electrocardiogram (EKG) showed sinus tachycardia, and bedside POCUS performed in the ED showed an ejection fraction of 40% using the modified Simpson method and revealed regional wall motion abnormalities (RWMA) in the midportion of the left ventricle (Fig. 1, see Videos S1, Supplemental Digital Content, http://links.lww.com/MD/N495 and S2, Supplemental Digital Content, http://links.lww.com/MD/N496, which demonstrate RWMA). This midventricular akinesia is indicative of the RWMA associated with SCMP. A thorough medical history and toxicology screening revealed no use of illicit drugs or substances that could contribute to drug-induced cardiomyopathy. This, along with the patient’s clinical presentation, alerted the ED physicians of the possibility of this diagnosis. The decision to admit the patient to the intensive care unit (ICU) was based on the presence of RWMA, which suggested potential coronary artery involvement. Elective coronary angiography was performed to investigate the underlying coronary pathology.

**Figure 2.**
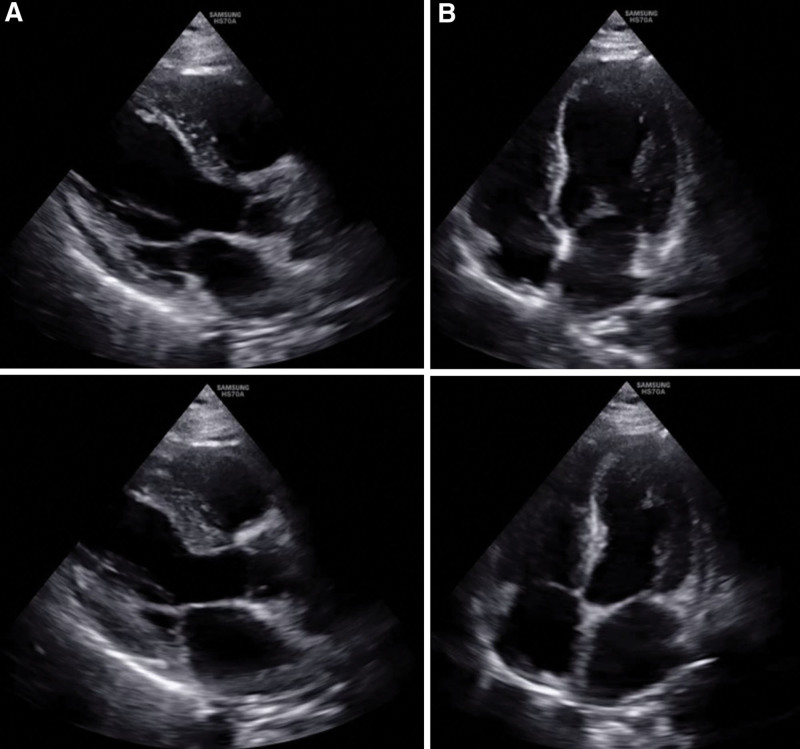
Follow-up POCUS of the end-diastolic and end-systolic phases performed 1 day after admission shows full recovery with a normalized ejection fraction and no regional wall motion abnormalities. (A) Parasternal long-axis view of the end-diastolic (upper) and end-systolic (lower) phases. (B) Apical 4-chamber view of the end-diastolic (upper) and end-systolic (lower) phases.

During the patient’s ICU stay, follow-up bedside POCUS showed improvement in the RWMA (Fig. 2, see Videos S3, Supplemental Digital Content, http://links.lww.com/MD/N497 and S4, Supplemental Digital Content, http://links.lww.com/MD/N498, which show improvement in the RWMA). The patient’s confusion subsided, and her cardiac enzyme levels normalized as follows: CK: 63 U/L, CK-MB: 2.59 ng/mL, and hs-TnT: 0.029 ng/mL (reference ranges: 26–192 U/L, 0.0–5.0 μg/L, and 0.000–0.014 ng/mL, respectively). Given the rapid clinical improvement and resolution of cardiac abnormalities observed via POCUS, the invasive coronary angiography procedure initially planned to investigate coronary issues was deemed unnecessary.

The patient showed significant symptomatic improvement, and she was discharged directly from the ICU without the need for further invasive diagnostic procedures.

## 3. Discussion

SCMP, commonly attributed to emotional stress, is a type of cardiac dysfunction known for its sudden onset after a severely stressful event.^[1–3,6]^ Among the various triggers of SCMP, recent reports have highlighted attempted suicide by hanging as a potential cause.^[7,8]^ This association presents a unique challenge in the ED,^[5,9]^ where differentiating SCMP from other coronary events can be particularly difficult. Traditionally, the confirmation of SCMP versus another coronary event has necessitated invasive procedures such as coronary angiography.^[1]^ However, the widespread adoption of bedside POCUS has highlighted it as a compelling alternative. POCUS allows for immediate, noninvasive assessment of cardiac function; this enables rapid diagnosis of SCMP, along with allowing ongoing monitoring of a patient’s condition without the risks or discomfort associated with more invasive diagnostic methods.^[4,5]^ This shift toward the use of POCUS in emergency and critical care settings underscores the need to reconsider traditional diagnostic pathways in favor of a less invasive, patient-centric approach to the diagnosis and management of SCMP, particularly in cases suspected to result from extreme stressors (i.e., attempted suicide by hanging).

This case demonstrates the utility of POCUS as an effective tool for emergency diagnosis and management of SCMP. POCUS facilitates quick assessment of a patient’s cardiac function, contributing to the delivery of timely and appropriate care. However, there are some limitations of POCUS that should be noted. Firstly, this procedure depends heavily on the operator’s skill and experience.^[4]^ Moreover, POCUS cannot provide detailed insights into a patient’s coronary artery status; this is accomplished using more invasive procedures such as coronary angiography.

The rapid resolution of SCMP observed in this case aligns with the findings of other case reports and studies, suggesting most patients with SCMP recover fully with supportive care. For instance, Templin et al highlighted the transient nature of SCMP and its favorable prognosis with conservative management.^[10,11]^ However, the present case report emphasizes the potential of POCUS in guiding the management of SCMP and reducing the need for invasive diagnostic procedures. Daily bedside follow-ups via POCUS minimize unnecessary diagnostic tests and shorten ICU stays; further, they enhance cost-effectiveness^[12,13]^ by streamlining patient care and reducing costs and the use of hospital resources.

The scientific rationale behind the noninvasive approach taken in this case was grounded in the understanding of SCMP pathophysiology. The condition involves a surge in stress hormones, leading to temporary myocardial stunning without the coronary artery blockages typical of myocardial infarctions.^[1,2]^ This knowledge, combined with the rapid improvement observed via POCUS, supported the decision against further invasive diagnostics. The key point highlighted by this case is the importance of integrating noninvasive diagnostic tools such as POCUS in the emergency management of SCMP, particularly in settings in which the clinical presentation may mimic more severe cardiac conditions.

This case contributes to the growing evidence supporting use of noninvasive diagnostic tools in emergency medicine and underscores the need for further research to optimize care for patients with SCMP. By demonstrating the efficacy of POCUS in diagnosing and managing SCMP after strangulation, this case advocates a paradigm shift toward noninvasive management strategies in emergency settings. While POCUS was instrumental in guiding the initial management in this case, it is important to note that SCMP is typically a diagnosis of exclusion. Confirmation through coronary angiography or cardiac MRI is generally recommended to rule out obstructive coronary disease. This case highlights the potential role of POCUS in the emergency setting but does not replace the need for comprehensive diagnostic protocols. Further studies are needed to evaluate whether POCUS alone can reliably diagnose SCMP.

## Author contributions

**Conceptualization:** Juho An.

**Data curation:** Juho An.

**Software:** Juho An.

**Writing – original draft:** Juho An.

**Writing – review & editing:** Juho An, Sung-Eun Lee.

## Supplementary Material


